# IoT Speech Recognition Application in Mass Sports Data Monitoring Based on Dynamic Adaptive Recommendation Algorithm

**DOI:** 10.1155/2022/8032571

**Published:** 2022-10-12

**Authors:** Meng Song

**Affiliations:** ^1^Tianjin University of Sport, Tianjin, China; ^2^Inner Mongolia University of Science and Technology, Inner Mongolia, China

## Abstract

In this paper, the existing dynamic adaptive recommendation methods are studied, which combine the practical application scheme of transforming the actual dynamic adaptive recommendation problem into user microblog information. After that, a dynamic adaptive weight fusion method is proposed and based on experimental verification, a real-time dynamic adaptive recommendation system is finally designed. The speech recognition of the Internet of Things takes natural language problems as the research object for a long time and takes the sound signal as the research topic. This paper analyzes the application of dynamic adaptive recommendation and Internet of Things speech recognition in mass sports data monitoring. The simulation results show that the system in this paper is convenient for users to monitor the exercise indicators in real time through the mobile client, and at the same time query the exercise historical data and compare the exercise data through the network terminal, thereby improving the exercise method and exercise load. Users can access the motion monitoring module and see the past floating state of motion parameters more intuitively than graphs, contains queries for metrics such as heart rate, body temperature, kinetic energy, pulse, and weight. Due to the diversity and complexity of people's differences, personal characteristics and business environments, sports data monitoring systems also need to be designed according to the scope of use. This paper analyzes the requirements for a motion data monitoring system and provides the system architecture design and basic data for producing detailed information for the system.

## 1. Introduction

This paper mainly proposes a dynamic adaptive recommendation method that meets the user's interest, content, and site operator's authority [1]. Based on the rearrangement of the adaptive recommendation method, the characteristics of the dynamic adaptive recommendation method are integrated [2]. In order to prove its practicability, it was verified by coding. After proving the effect of the method, this paper designs and develops a personalized prototype system. The experimental environment of this article is (1) hardware configuration: Intel(R) I5-3110M3.2 GHz, 12G memory; (2) operating system: Win764 bit; (3) development environment: Eclipse, MySQL, Navicat, editolus R2012. At this stage, most of the Internet of Things speech recognition technologies are based on statistical models. From the principle of speech generation, speech recognition can be roughly divided into two parts: speech layer and language layer [3]. The difficulty of current Internet speech recognition technology can be solved from the aspect of speech characteristics. Audio model is the most basic model that represents audio information, and it is also an important part of the system [4]. The type of training unit directly depends on the method of feature extraction. Feature extraction requires the extraction function not only to effectively reduce the data volume of the digital audio signal, but also to ensure the validity of all the original audio information [5]. However, due to the serious deficiencies in the development system of mass sports at this stage, the specific manifestations are incomplete basic facilities, imperfect organizational structures, insufficient funds, lack of management talents, and lack of “work is sports” [6]. On this basis, we can take advantage of the favorable promotion of new construction to actively study the development path of mass sports in the field of construction, which has important significance in practical applications [7]. The sports data monitoring system server must provide real-time services for many sports users [8]. In order to realize real-time data monitoring, the system server provides stable and efficient features with low latency. At the same time, online operations are supported. By analyzing two traditional synchronization models, it is difficult for the traditional synchronization model to meet the scalability of complex network services [9]. The SEDA concurrency architecture model can flexibly adapt to system requirements and expansion, and combines the advantages of multithreading and event-driven that can maintain excellent performance in the environment. This will simplify the development of the system server, while also increasing the speed of the server, which contributes to the performance of the entire system [10]. Provides a strong foundation to optimize and promote server performance.

## 2. Related Work

The literature introduces the research content of the sports health evaluation model and explains the concept of sports health evaluation. Due to the complexity and randomness of the movement process, we can use the Markov statistical model for analysis. Next, the basic theory of the MARKP model is described, and the characteristics of the Markov model are analyzed [11]. Finally, for sports health assessment and various requirements of the group, an exercise model of health assessment is proposed. The literature introduces a real-time monitoring system for data collection hardware equipment, user equipment data, and sports data [12]. The data collection hardware equipment can be used to obtain real-time user data and display sports data. Among them, the data collection device uses 3D acceleration sensors to analyze the characteristics of people participating in the movement. These data are converted into a base station, and the base station sends specific data to the system communication service memory, combining the characteristics of the user's body and the energy consumption of the exercise, and finally sends the obtained data to the mobile monitoring terminal [13]. The literature introduces the operation time and energy consumption data obtained from the exercise data real-time monitoring system and proposes a health assessment model to explain the data consumption and personalized exercise to achieve the purpose of providing recommendations [14]. The literature analyzed the characteristics of energy consumption and individual height and weight during exercise, and proposed a health evaluation model for sports population [15]. The purpose is to eliminate the differences caused by individuals in sports, make an objective evaluation of the athletes, and give a reasonable evaluation effect to the sports team. The literature describes the basic principles of neural networks and the basic theories of audio signal processing [16]. First of all, based on the principle of speech recognition, the speech recognition has been improved. Including digital sampling of audio signals and digital sound signal analysis, while spectrum analysis technology is suitable for signal content analysis and extraction. The literature introduces the back propagation network model and the deep belief network model, and gives some opinions and suggestions, many of which still need to be in-depth research and testing. In terms of speech processing, the samples used are collected in an ideal indoor environment. In practice, the samples may have obvious noise. Therefore, the influence of noise should be eliminated first in the experiment process to improve practicability.

## 3. Theoretical Basis of Dynamic Adaptive Recommendation and Internet of Things Speech Recognition

### 3.1. Dynamic Adaptive Recommendation

In this article, taking into account the degree of decline in the time value, it is divided into the following processes:

1. Based on the original data, calculate sports popularity and time decay factors, and save them in their respective popularity databases and damping factor databases
2. Use the neighborhood method to calculate the user motion similarity matrix and store it in the neighborhood method similarity database
3. The loss function is defined by the matrix factorization method and stored in the loss function library of the matrix factorization method, and the processed data is used as the score value of the sports user
4. The adjacent optimizer consolidates the similarity between flow motions in two stages to optimize the neighborhood method
5. Fuse the loss function and the results of the 4 steps according to the initial ratio
6. Combine the results of the time loss coefficient with the dynamic merge module to generate a list of suggestions
7. Dynamically train the weighting factor according to the call of the recommendation result to find the best factor value

**Figure 1:**
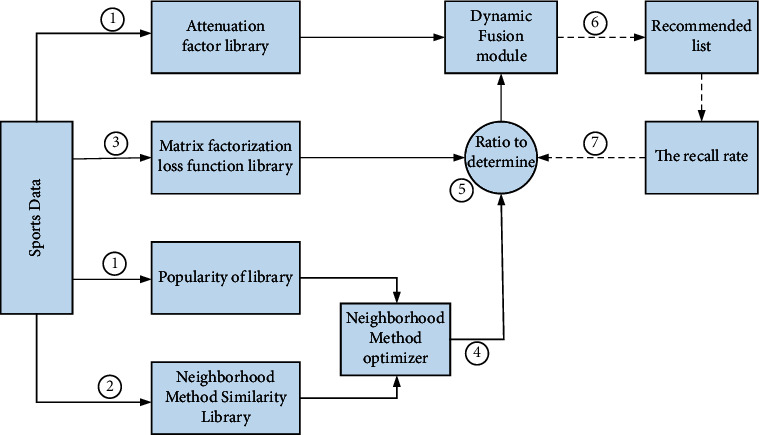
Dynamic adaptive fusion recommendation system framework.

Figure 1 shows the recommendation system architecture of the dynamic adaptive fusion algorithm.

Through calculation and analysis, the square root of these types of exercise sample sizes can reduce missing data and improve the accuracy of recommendations. The improved popular formula is as follows:

(1) $$\begin{array}{c} {pop\;}_{j}=\left\{\begin{array}{c} \frac{\left|N\left(j\right)\right|}{\sum_{j\in pp}\left\|N\left(j\right)\right\|},Popularity⩾200,\\ \frac{\left|N\left(j\right)\right|}{\sum_{j\in pop<200}\sqrt{\left|N\left(j\right)\right.}},Popularity<200\text{.} \end{array}\right. \end{array}$$

All developments are time-sensitive, so this article introduces the concept of time fading. Suggestions for concave time decay function is as follows:

(2) $$\begin{array}{c} f\left(x\right)=\varphi^{x}\text{.} \end{array}$$

Popular sports have long-term effects, while low-popular sports have temporary effects, so we first define the time frame function and use it as an exponential variable.

(3) $$\begin{array}{c} f_{ts}=-\left(T_{c}-T_{0}\right)\text{,}\\ TD_{j}=e^{f_{ts}}\times \frac{1}{pop_{j}}\text{.} \end{array}$$

The neighborhood method is used to calculate the similar relationship between users and sports according to formulas.

(4) $$\begin{array}{c} W_{ij}=\frac{\left|N\left(i\right)\cap N\left(j\right)\right|}{\sqrt{\;|\;N\left(i\right)}\|N\left(j\right)\;|\;}\text{.} \end{array}$$

The matrix factorization technique uses the following formula to optimize the function of the power difference.

(5) $$\begin{array}{c} f_{loss\;}=\overset{n}{\underset{i=1j=1}{\sum }}\overset{m}{\underset{i=1}{\sum }}{\left(R-u_{i}^{T}\times v_{j}\right)}^{2}\text{.} \end{array}$$

First, we define a similarity consistency function to ensure the consistency of the similarity matrix between users and sports obtained in the two calculations.

(6) $$\begin{array}{c} f_{smc-u}=\overset{n}{\underset{i=1}{\sum }}{\left(\begin{array}{c} \underset{u_{p}\in KNN\left(u_{i}\right)}{\sum }W_{ip}\times u_{p}\\ \left|KNN\left(u_{i}\right)\right| \end{array}\right)}^{2}\text{,}\\ f_{smc-v}=\overset{m}{\underset{j=1}{\sum }}{\left(\begin{array}{c} \underset{v_{jq}}{\sum }\times v_{q}\\ v_{j}-\frac{v_{q}\in KNN\left(y_{j}\right)}{\left|KNN\left(v_{j}\right)\right|} \end{array}\right)}^{2}\text{.} \end{array}$$

Whether users like sports and whether they are affected by popularity, so the optimization by multiplying the popular pop_j_ by the f_smc-v_ function is based on the proximity method.

(7) $$\begin{array}{c} f_{smc}=f_{smc-v}\times pop_{j}+f_{smc-u}\text{.} \end{array}$$

Combining adaptive alpha weighting with time saving, used for time-based, adaptive *f*_taf_ mixing function, based on adjacent matrix transformation methods and techniques.

(8) $$\begin{array}{c} f_{taf}=\left(\gamma \times f_{smc}+\left(1-\gamma \right)\times f_{loss}\right)\times TD_{j}\text{.} \end{array}$$

Finally, we introduced a normalization function to prevent the dynamic adaptive recommendation algorithm from affecting the noisy data, so as to obtain the final objective function.

(9) $$\begin{array}{c} f_{MFDA}=f_{taf}+\lambda \times \left({||u_{i}||}^{2}+{||v_{j}||}^{2}\right),\\ u_{i}=\frac{Nei\;\left(u\right)+\left(1-\gamma \right)\times TD_{j}\times V\times R_{i}}{\gamma \times TD_{j}+\left(1-\gamma \right)\times TD_{j}\times V\times V^{T}+\lambda }\text{,}\\ v_{j}=\frac{Nei\;\left(v\right)\times pop_{j}+\left(1-\gamma \right)\times TD_{j}\times U\times R_{j}}{\gamma \times TD_{j}\times pop_{j}+\left(1-\gamma \right)\times TD_{j}\times U\times U^{T}+\lambda }\text{.} \end{array}$$

Using the optimal solution of the alternating least square method, the prediction score matrix is finally obtained.

(10) $$\begin{array}{c} Nei\left(u\right)=\frac{\gamma \times TD_{j}\times W_{ip}\times u_{p}}{\left|KNN\left(u_{i}\right)\right|}\text{,}\\ Nei\left(v\right)=\frac{\gamma \times TD_{j}\times W_{jq}\times v_{q}}{\left|KNN\left(v_{j}\right)\right|}\text{.} \end{array}$$

In the simple addition fusion algorithm, the ratio of the neighbor method matrix decomposition method is 0.5. For comparison, the adaptive weighting factor of MFDA is fixed at 0.5. These three age records are applied to the FSWA and MFDA algorithms. The specific results are shown in Table 1.

### 3.2. Internet of Things Speech Recognition

Sound waves are transmitted from the outer ear to the machine and converted into mechanical energy. When the last sogol (forged bone) in the cochlea moves, the flow of cochlear fluid generates traveling waves from the basement membrane. The impulse response of the cochlear basement membrane is represented by the function *ψ*(*t*).

The response integral is zero.

(11) $$\begin{array}{c} \int_{-\infty }^{+\infty }\psi \left(t\right)dt=0\text{.} \end{array}$$

Mean square integrable or finite energy.

(12) $$\begin{array}{c} \left.\int_{-\infty }^{+\infty }\right|\psi \left(t\right){|}^{2}dt<\infty \text{.} \end{array}$$

Satisfy,

(13) $$\begin{array}{c} \int_{-\infty }^{+\infty }\frac{{\left.\Psi \left(\omega \right)\right|}^{2}}{\omega }d\omega =C\text{,}\\ \Psi \left(\omega \right)=\int_{-\infty }^{+\infty }\psi \left(t\right)e^{-j\omega t}d\omega \text{,} \end{array}$$

where *f*(*t*) can all be mean square integral functions. The auditory conversion that converts the impulse response *ψ*(*t*) of the base film to *f*(*t*) is defined as follows:

(14) $$\begin{array}{c} T\left(a,b\right)=\int_{-\infty }^{+\infty }f\left(t\right)\frac{1}{\sqrt{a}}\psi \left(\frac{t-b}{a}\right)dt\text{.} \end{array}$$

The above formula can also be written as follows:

(15) $$\begin{array}{c} T\left(a,b\right)=\int_{-\infty }^{+\infty }f\left(t\right)\psi_{a,b}dt\text{,}\\ \psi_{a,b}\left(t\right)=\frac{1}{\sqrt{a}}\psi \left(\frac{t-b}{a}\right)\text{.} \end{array}$$

This ensures that all values of *α* and *b* have the same energy.

(16) $$\begin{array}{c} \int_{-\infty }^{+\infty }{\left|\psi_{a,b}\left(t\right)\right|}^{2}dt=\left.\int_{-\infty }^{+\infty }\right|\psi \left(t\right){|}^{2}dt\text{.} \end{array}$$

Definition formula of cochlear basilar membrane filter.

(17) $$\begin{array}{c} \psi_{a,b}\left(t\right)=\frac{1}{\sqrt{a}}{\left(\frac{t-b}{a}\right)}^{\alpha }\;\exp\;\left(-2\pi f_{L}\beta \left(\frac{t-b}{a}\right)\right)\cos\;\left(2\pi f_{L}\left(\frac{t-b}{a}\right)+\theta \right)u\left(t\right)\text{.} \end{array}$$

The demand for inverse conversion comes from the reconstruction of decomposed signals such as music synthesis or noise reduction. Similarly, the original signal can be restored if it is inversely transformed.

(18) $$\begin{array}{c} f\left(t\right)=\frac{1}{C}\int_{a=0}^{+\infty }\int_{b=0}^{+\infty }\frac{1}{\left|a{|}^{2}\right.}T\left(a,b\right)\psi_{a,b}\left(t\right)dadb\text{.} \end{array}$$

Discrete-time auditory positive transformation.

(19) $$\begin{array}{c} T\left[a_{i},b\right]=\overset{N}{\underset{n=0}{\sum }}f\left[n\right]\frac{1}{\sqrt{\left|a_{i}\right|}}\psi \left[\frac{n-b}{a_{i}}\right]\text{.} \end{array}$$

Discrete-time inverse auditory transformation.

(20) $$\begin{array}{c} \tilde{f}\left[n\right]=\frac{1}{C}\overset{o_{k}}{\underset{a_{i}=a_{1}}{\sum }}\overset{N}{\underset{b=1}{\sum }}\frac{1}{\left|a_{i}\right|}T\left[a_{i},b\right]\psi \left[\frac{n-b}{a_{i}}\right]\text{,}\\ h\left(a,b\right)={\left[T\left(a,b\right)\right]}^{2}\forall a,b\text{.} \end{array}$$

In the next step, the output *S*(*i*, *j*) of each hair cell represents the relevant neural spike in the center frequency response of the current filter.

(21) $$\begin{array}{c} S\left(i,j\right)=\frac{1}{d_{i}}\overset{l+d_{-}-1}{\underset{b=1}{\sum }}h\left(i,b\right),l=1,L,2L,\cdots ;\forall i,j\text{.} \end{array}$$

The output of hair cells is converted into energy-perceived loudness through a nonlinear cube root loudness function.

(22) $$\begin{array}{c} y\left(i,j\right)={\left[S\left(i,j\right)\right]}^{1/3}\text{.} \end{array}$$

Table 2 shows the frequency division under the Bark scale:

Assuming that the original ignition is *x*(*n*), 0 < *n* < *N*, the auditory conversion coefficients *T*(*a*, *b*) of *m* channels and *N* points after auditory conversion are obtained. 1 ≤ *a* ≤ *m*, 1 ≤ *b* ≤ *N*, where *α* represents different channels corresponding to different center frequencies and *b* represents different positions at different times, depending on the audio signal processing result.

## 4. Design and Practical Application of Mass Sports Data Monitoring System

### 4.1. Demand Analysis of Mass Sports Data Monitoring System

Before starting the topic, we determined that the system can meet the needs of three users and offline surveys. (1) We can monitor the user's exercise index in real time. (2) The user's exercise index can be judged. (3) We can provide healthy exercise guidance according to the changes of users' exercise parameters.

The client mainly refers to the Android application. After the user registers with the client, if the login is successful, the APP will enter the basic interface of the function. The main interface of the APP is divided into five functional modules in the setting module: exercise equipment connection, exercise indicator monitoring, self-health assessment, healthy exercise guide, and user's health file.

**Figure 2:**
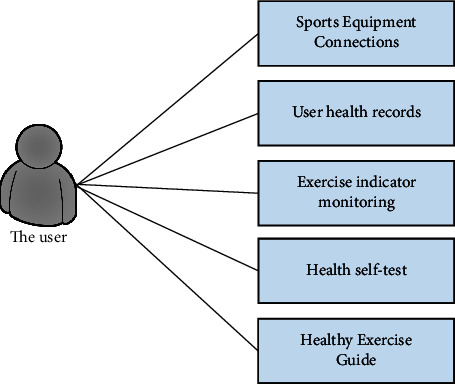
Client use case diagram.

**Figure 3:**
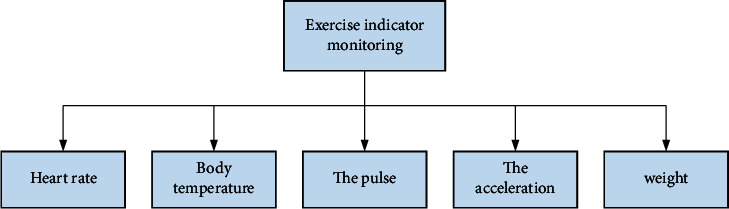
Use case diagram of sports indicator monitoring.

1. Connection of exercise equipment: to monitor the flow indicator, the user must connect the customer to a smart portable exercise monitoring device. The user clicks the “Connect Device” button to add a device, and the client's Bluetooth must be turned on before the device can be bound.
2. Monitoring of exercise indicators: the functional exercise monitoring module is a module for real-time monitoring of user exercise data. This module mainly displays the flow data of the portable exercise monitor on the smart terminal and records the heart rate, body temperature, pulse, exercise speed, weight, and other exercise indicators. Users can monitor their movements in real-time anytime and anywhere, which is useful for accurately understanding their movements. Client use case diagram as shown in Figure 2.
3. When the user clicks “monitor exercise,” the interface will take us to the “exercise data view” page. We can see the movement trend chart from the zero point of the week to the present. The data is updated every minute and the latest data is displayed in digital form in the upper left corner of the trend graph. And when we select an indicator, the indicator name will turn blue, and the trend chart corresponding to the indicator will be displayed. When you move the mouse to a specific point on the trend graph, the data floating layer of the selected indicator at that point will be displayed. The system will provide personalized health exercise guidance based on exercise data. As shown in Figure 3.
4. Self-health assessment: the functional module consists of three parts: basic information, health status, and exercise habits. The basic information module records the user's gender, age, weight, and other information; exercise habits: the user's exercise intensity, daily exercise time, etc. If the information recorded in the user's health file is different from the user's actual information, you can manually change it.
5. Healthy exercise guide: the system provides a way to obtain multiple sets of self-health questionnaires from various aspects. The system will also identify users' anxiety and depression levels based on user problems and provide them with detailed health improvement plans.
6. User's health file: daily articles on various healthy exercise guidelines, healthy diet guidelines, etc., will be published on the APP homepage. Improve users' health awareness, develop a healthy lifestyle, and prevent chronic diseases at the source.

After the user completes the registration work from the mobile terminal, he can directly log in to the network terminal. If the user is not registered, the registration process must be completed, and the mobile terminal and the network terminal will synchronize user information. If the user is not logged in, he is not authorized to use some function modules. Users can input personal sports information through mobile devices or web devices to change their sports information. This information will be applied after the user clicks “Save,” and the user's sports information on their mobile devices will be updated at the same time. If the practice data is different from the actual value, you can click the “Edit” or “Delete” button to perform the task.

When we click on “Sports Trends,” the trend chart of the day the user selects the indicator will be displayed by default. The trend chart can display up to two indicators at the same time. When we move the mouse to a specific point in time, we will see the data drawing layer, and the indicator is now selected. According to the user's health report, the system uses a simple algorithm to obtain the user's health score, evaluate the health level, provide health notifications, and personalize the user based on the level, and we provide a reasonable recovery set plan.

Customers of this system are assigned to Android smartphones with different user groups. The display that can carry the human motion parameters is easy to display and does not require complicated work. The web-side motion parameter management cloud platform is almost distributed on every home computer. Users can monitor their movement anytime and anywhere, and if they find an abnormality, they can stop the movement. The system uses MySQL (a scalable relational database to store data) and uses HTTP communication for data communication between the client and the server to ensure data accuracy. The wearable motion parameter monitor and the client can use the Socket data communication method to synchronize the motion index data in real time and download it to the client. Scalability refers to the data transmission capacity of the system, and the coordination of single or system-wide functional modules can meet the conversion needs. The system uses the highly scalable Android platform and JavaEE Web platform, and the database uses the relational database of MySQL and SQLite, so the system can perform read-write isolation and data fragmentation processing, which can improve the overall throughput.

### 4.2. System Architecture Design

**Figure 4:**
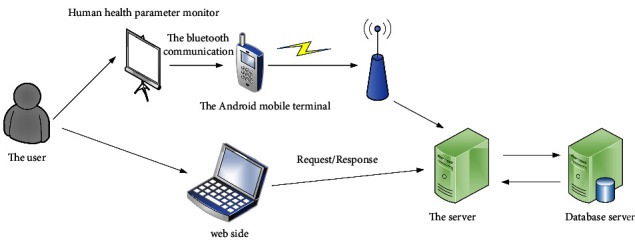
System overall architecture diagram.

The system is based on the Android platform and configures wearable health monitoring through various sensors to collect the person's heart rate, temperature, pulse, acceleration, and other exercise parameters, and performs exercise detection through the Bluetooth module. The Android client allows users to view and evaluate their health level through the Web health management cloud platform to monitor their exercise status in real time. The system consists of three parts: wireless sensor module, intelligent terminal module, and server module. Among them, the wireless sensor module is a smart module and a Bluetooth module, the smart terminal module is a mobile phone and an Android cloud platform, and the server module is a background server of the database. The sports health monitor collects data about the user's exercise, and the server receives the traffic data measured by the sports health monitor, analyzes it, and responds to mobile phone customers' requests. The Android client and the practice display communicate with data via Bluetooth. After activating the display, it is waiting to establish a Bluetooth connection with the Android phone. When two Bluetooth devices are connected to the same Bluetooth device RFCOMM channel, the mobile phone and the monitor normally establish a Bluetooth connection, the mobile phone obtains the motion monitor data, the server receives the connection request from the client, and then establishes the interactive thread data logic with the client. The whole system architecture is shown in Figure 4.

After the portable human motion monitor communicates with the Android phone through the Bluetooth module and is properly configured through AT commands, the Android smart phone will collect traffic information on the motion monitor. The built-in Bluetooth portable exercise parameter monitor for mobile phones can communicate data through Socket. After the Android mobile phone receives the exercise instruction data, it will analyze the data, save it, and display it in a certain format. It is a waveform diagram of the Android mobile APP interface.

Android XML development use layout files to design the interface, and each functional module uses activity classes to interact with users. The system is mainly divided into a motion parameter module and a maintenance module. The motion control module includes a heart rate module, a body temperature module, a kinetic energy module, and a heart rate module. There are four service modules: save practice data, process data, completeness warning, and manage practice information, and then click practice information.

### 4.3. Functional Module Design

#### 4.3.1. The Layout of the Registration Page

When the user uses the system application for the first time, user registration is required. When designing the UI interface of the “user registration” functional module, first create the layout file register.xml corresponding to the Activity, and then define the relative layout on the other side. EditText controls and buttons will be created in this layout. The EditText control is used to enter user information. Click the “Button” button to send the registration information entered by the external user. Then, generate another interface data.xml for receiving and sending data. The layout of the interface is relatively simple. Eight textViews have been added, and the registration form will display username, password, confirm password, gender, e-mail, and mobile phone. User information (such as b, Numbers, and real-time verification codes) will not be displayed in this interface of the application.

#### 4.3.2. The Specific Realization of the Registration Function

After creating the UI interface, create the RegisterActivity class inherited from the Activity class and implement the OnClickListener interface to cover onClick, where the getText method represents the registration information input by the user. Call the network interface class (requestapidat a) to create a getregistdata method, which interacts with the interface to create a user name, password, real name, gender, mobile phone number, e-mail, and other information entered by the user. Regardless of the login or registration function, it is very dangerous to send the password in plain text when connected to the network. The system uses the MD5 information digest algorithm combined with BASE64 to encrypt the password and calls the MD5 digest method to obtain a partial array of encrypted words.

This project is a network-side personal health exercise management that allows users to manage personal exercise parameters on various platforms (mobile and PC) to achieve personal health exercise management on multiple platforms. The system uses the open-source framework Spring, SpringMVC, and Mybatis to design a webchuk health exercise management cloud platform. The health exercise management cloud platform in the function module, registration module, and login module of the Weptuk system is divided into user health. The specific implementation of each functional module will be described in detail below.

If we are not familiar with the Webside health exercise management cloud platform, we need to register by clicking the register button at the top right of the homepage. When the user clicks the registration button, a registration mode dialog box will pop up, and the information that needs to be entered on the registration page includes user name, password, gender, e-mail, and mobile phone number. The design of the registration interface mainly combines Spring mvc and JSP technology. In order to prevent unauthorized user input and malicious hacker attacks, the system introduces a verification mechanism, which regularly checks the information entered by the user on the register.jsp page. For example, when a user enters his mobile phone number on the registration page, if the input format or length does not match the regular expression format, the mobile phone number is entered incorrectly. At this time, a message will be displayed on the registration page showing the relevant mobile phone number and information that does not meet specifications. After entering the registered mobile phone number correctly, click “Get Confirmation Code,” the page will display a 60-second countdown to start receiving the SMS verification code. The SMS verification function is an HTTP interface provided by the third party sendSmsByHTTP. It is used to send Jixintong calls using the SMS platform Ali Da and sends the SMS template in the AlibabaSmsUtils tool class, which sends short messages.

After the user successfully logs in, click “Healthy Sports File” to view past sports data. In this system, the exercise data of the health exercise management cloud platform is directly called from the database, and all these data are sent to the mobile client as exercise index data. The exercise index data is collected by the user using a wearable human exercise parameter monitor. The monitoring will be saved. The database uses paging technology to display a system generated from a large amount of relative exercise data measured at different time periods in a healthy exercise file.

### 4.4. Database Design

The exercise data collected by the Android traffic monitor is retrieved via Bluetooth, and the exercise data is maintained locally and online to ensure that the data on the Android page is synchronized with the data on the server page. As shown in Table 3.

For Android, S'Lite is a lightweight database optimized for mobile devices. The system has developed an Android S'Lite database named joy Health, which contains five tables. (See Table 4)

## 5. Conclusion

In this course, we will design and implement a human health exercise management system based on Java Web and Android devices, and use cloud data processing technology to upload and save exercise parameters to the cloud. It is convenient for users to monitor exercise indicators in real time through the mobile client, and at the same time query exercise history data and compare exercise data through the network terminal, thereby improving exercise methods and exercise load, and avoiding physical discomfort caused by exercise. Scholars continue to study neural networks and audio signal processing. In this article, we use neural networks for speech recognition to conduct a more detailed study, so as to provide a preliminary exploration of the above problems. Deep learning is a new subfield of machine learning in the past few years, which focuses on NN model configuration and unsupervised learning using multiple layers of nodes. These detailed neural networks can better handle complex intelligence problems, and the way to process network model information is to imitate the human brain and can be used for speech recognition.

## Figures and Tables

**Table 1:** The relationship between times, weighting factors, and RMSE in different algorithms.

Index	10 to 29 years old		30 to 49 years old		50 to 69 years old	
	FSWA	MFDA	FSWA	MFDA	FSWA	MFDA
Training times weight factor RMSE	4	5	3	3	5	4
	0.4	0.15	0.2	0.34	0.7	0.74
	1.283	0.812	1.221	0.731	1.218	0.793

**Table 2:** Bark scale filter center frequency division.

Filter number	Center frequency (Hz)
1	50
2	150
3	250
4	350
5	450
6	570
7	700
8	840
9	1000
10	1170
11	1370
12	1600
13	1850
14	2150
15	2500
16	2900
17	3400
18	4000

**Table 3:** Mobile phone user information table (table name: user).

Field	Type of data	Field description
Id	Int	Data table auto-increment field
Username	Varchar	Registered user name
Password	Varchar	Password
Realname	Varchar	Actual name
Sex	Varchar	Gender
E-mail	Varchar	Register email
Phone	Varchar	Register mobile phone number
Date	Varchar	Registration time field
Is updated	Int	Whether to update to the server

**Table 4:** Heart rate measurement results of mobile phone users (table name: heart).

Field	Type of data	Field description
Heart_id	Int	Data table auto-increment field
Username	Varchar	Username field
Heart_value	Varchar	Weight measurement result field
Date	Varchar	Update time field
Is updated	Int	Whether to update to the server

## Data Availability

The data used to support the findings of this study are available from the corresponding author upon request.
